# Effect of Access Channel Sealing and Superstructure Material on Abutment–Implant Screw Stability After Cyclic Loading: A Comparative In Vitro Study

**DOI:** 10.3390/ma19081635

**Published:** 2026-04-19

**Authors:** Zeynep Irkeç, Ayben Şentürk, Kaan Orhan

**Affiliations:** 1 Department of Prosthetic Dentistry, Faculty of Dentistry, Lokman Hekim University, Ankara 06510, Turkey; 2 Department of Prosthetic Dentistry, Faculty of Dentistry, Ankara University, Ankara 06100, Turkey; dr.dt.aybensenturk@gmail.com; 3 Department of Dentomaxillofacial Radiology, Faculty of Dentistry, Ankara University, Ankara 06100, Turkey; knorhan@gmail.com; 4 Department of Oral Radiology, School and Hospital of Stomatology, Cheeloo College of Medicine, Shandong University, Jinan 250012, China

**Keywords:** ceramics, composite resins, dental implants, dental implant-abutment design, screw loosening, silicones

## Abstract

**Highlights:**

- Access channel sealing material significantly affected RTV after cyclic loading.
- A material-dependent interaction was observed, indicating that the effect of sealing material varied according to the superstructure material.
- Silicone-based sealing improved torque maintenance in zirconia restorations, whereas no significant differences were observed in resin nano-ceramic groups.
- Superstructure material alone did not significantly influence RTVs.
- The findings highlight the importance of considering implant restorations as a biomechanical system with interacting components.

**Abstract:**

Background: Screw loosening remains a common mechanical complication in implant-supported restorations; however, the combined effect of sealing and superstructure materials on abutment screw stability warrants further investigation. Methods: This study evaluated the influence of access channel sealing material and superstructure material on abutment–implant screw stability after thermomechanical cyclic loading. Forty-eight Straumann analog–abutment assemblies restored with monolithic zirconia or resin nano-ceramic (Cerasmart) crowns were assigned to two sealing protocols: Polytetrafluoroethylene (PTFE) + composite or polyvinyl siloxane (PVS) putty (n = 12). After 750,000 off-axis cycles, reverse torque values (RTV) were analyzed using two-way analysis of variance (ANOVA) and Tukey’s HSD, with effect sizes calculated (α = 0.05). Results: A significant interaction between restorative material and sealing protocol was observed (*p* = 0.0170; η^2^ = 0.116). Superstructure material showed no significant influence on RTV (*p* = 0.8368), whereas sealing protocol had a significant main effect (*p* = 0.0499). RTVs were highest for zirconia + PVS putty (36.33 ± 4.53 Ncm) and lowest for zirconia + PTFE (29.32 ± 6.30 Ncm), while the Cerasmart groups showed intermediate values. Post hoc analysis confirmed higher RTV for zirconia + PVS compared with zirconia + PTFE (*p* = 0.0138). Conclusions: Access channel sealing materials showed a material-dependent influence on abutment screw stability. Silicone-based sealing improved torque maintenance in zirconia, indicating that rigid restorative materials may be more sensitive to sealing material selection. In contrast, Cerasmart showed comparable RTV regardless of sealing protocol, suggesting that resilient restorative materials may reduce the influence of sealing on preload maintenance.

## 1. Introduction

The long-term success of implant-supported restorations depends not only on osseointegration but also on implant–abutment stability, as this screw-mediated interface plays a critical role in resisting occlusal loading [1]. Despite high survival rates, mechanical complications remain clinically relevant under prolonged function [2,3], with screw loosening being the most frequently reported event (≈2.1%) in implant-supported restorations. However, reported rates may vary depending on clinical conditions, prosthetic design, and follow-up duration, including differences between single crowns and fixed partial restorations as well as variations in observation periods across studies [4]. Furthermore, this complication is also considered a potential precursor to screw fracture [1,3,5]. Accordingly, ensuring implant–abutment stability is essential for the long-term mechanical reliability of implant-supported restorations.

Given the importance of implant–abutment stability, preload refers to the internal stress generated within the abutment screw during initial tightening, creating a clamping force at the implant–abutment interface [1,6,7,8]. Although primarily dependent on the applied torque, it may also be influenced by factors such as screw design, material properties, and passive fit [1,6,9,10,11]. Early preload reduction may occur within minutes due to the settling effect, with reported reductions ranging from 2% to 10% [12]. Accordingly, Siamos et al. (2002) recommended retightening 10 min after the initial torque application [13], a protocol also supported by other studies [7,11,14]. Because preload is a key determinant of screw stability, its preservation is critical for maintaining joint integrity under functional loading [7,15]. Therefore, any factor that compromises preload maintenance may directly increase the susceptibility to screw loosening under clinical conditions.

With respect to the number of units involved, previous studies have reported a higher incidence of screw loosening in single-tooth restorations [2,11,15], with 5-year rates ranging from 5.8% to 12.7% [16,17]. Notably, screw-retained prostheses have been shown to exhibit higher loosening rates (32 ± 0.3%) compared with cement-retained restorations (9 ± 0.2%), a difference attributed to reduced passive fit in the former design, which may influence preload distribution [18]. Such variability in reported loosening rates may be attributed to differences in prosthetic design and restoration configuration, such as single crowns versus fixed partial restorations, as well as variations in observation period, study design, and clinical conditions, all of which should be considered when interpreting these values. Angulated screw-retained restorations have been reported to exhibit particularly increased susceptibility to loosening [19]. Although cement-retained restorations may offer improved esthetics and fewer mechanical complications, limitations in retrievability remain a concern [19,20]. Collectively, these findings highlight that both prosthetic design and retention strategy play a decisive role in the development of screw-related mechanical complications.

Building on the influence of retention strategy on implant–abutment stability, both solid abutments and abutments with a screw access channel may be used in cement-retained restorations [21]. When present, this channel may be sealed using materials such as polytetrafluoroethylene (PTFE), composite resin, temporary filling materials, or polyvinyl siloxane (PVS) [22]. In clinical practice, the most common approach involves placing PTFE over the screw head [23], followed by a composite resin layer [24]. Furthermore, da Rosa et al. (2024) reported that composite sealing may improve the mechanical performance of lithium disilicate restorations compared with unfilled designs, whereas PTFE alone showed less favorable outcomes [21]. Beyond their sealing function, these materials may also play a biomechanical role in load distribution within the implant–abutment complex. Previous research has primarily examined the effects of these materials on marginal adaptation, retention, and microbiological sealing [3,7,23,25]. However, evidence regarding the influence of screw access channel sealing materials on abutment screw fatigue resistance and loosening warrants further investigation [21,22].

In addition to sealing strategies, the choice of superstructure material, such as lithium disilicate, zirconia, and polyetheretherketone (PEEK), represent another key determinant of implant biomechanics [26]. In a study, restorations fabricated from materials with a higher elastic modulus exhibited reduced deformation and transmitted greater loads to the implant [27]. Although resin-based materials may provide more favorable stress absorption, their lower resistance to moisture and mechanical stress may affect long-term stability. Accordingly, both superstructure material properties and access channel sealing strategies should be considered as interacting factors influencing implant–abutment stability [15].

Beyond the initial preload, the second stage of screw loosening is associated with the type and magnitude of external functional loads [7,11,15]. When preload falls below a critical threshold, micromovement may occur at the implant–abutment interface and has been reported to range between 1.52 and 94 µm in a previous study [28], leading to reduced reverse torque values [7,11]. Accordingly, previous studies have highlighted parafunctional habits and prosthetic misfit, and proposed occlusal and design modifications to minimize screw loosening [29,30]. In this context, cyclic loading has been widely used to simulate masticatory function under controlled in vitro conditions [10]. Thermal aging provides an additional laboratory model to simulate intraoral conditions and may promote resin cement degradation, thereby influencing load transfer at the abutment–implant interface according to the restorative material [31]. These considerations underscore the importance of evaluating screw stability under simulated functional conditions that closely reflect the intraoral environment.

As a measure of screw joint stability, the reverse torque value (RTV) is widely used to assess residual preload following fatigue loading [8]. Accordingly, this study aimed to evaluate the effects of screw access channel sealing material and superstructure material on abutment–implant screw stability after thermomechanical cyclic loading by measuring RTVs. Previous studies have primarily investigated the effects of access channel sealing materials on retention, microleakage, and marginal adaptation [3,7,23,25], but their potential influence on abutment screw stability warrants further investigation. In addition, although the biomechanical behavior of different restorative materials has been widely examined [26,27], the possible interaction between superstructure material and access channel sealing strategy in relation to screw stability has not been clearly established. Therefore, the combined biomechanical effect of these two variables on screw stability remains unclear, representing a critical gap in the current literature. To address this, a comprehensive evaluation of both their individual and interactive effects is necessary to clarify their role in maintaining implant–abutment stability under functional loading conditions. Accordingly, the present study was designed to address this gap by evaluating not only the individual effects of these variables but also their combined influence on RTV under simulated functional conditions. It was hypothesized that both the access channel sealing material and the superstructure material would influence RTV after thermomechanical cyclic loading, and that this effect would be material-dependent. Specifically, restorative materials with a higher elastic modulus were expected to transmit greater stresses to the implant–abutment complex, potentially resulting in lower RTVs, whereas more elastic silicone-based sealing materials were anticipated to improve stress distribution within the access channel and contribute to higher RTVs.

## 2. Materials and Methods

This in vitro study was conducted at the Faculty of Dentistry, Ankara University, Ankara, Turkey. As no human or animal subjects were involved, ethical approval was not required.

An a priori power analysis was conducted using G*Power software (version 3.1.9.7; Heinrich Heine University, Düsseldorf, Germany) to determine the minimum sample size required for the two-way ANOVA design with two independent factors (superstructure material and sealing material). Assuming a medium effect size (f = 0.25), an alpha level of 0.05, and a statistical power of 0.80, the analysis indicated that a minimum of 44 specimens would be required. Therefore, the total sample size of 48 specimens used in the present study was considered sufficient to detect statistically meaningful differences among the experimental groups, while allowing for potential specimen loss.

### 2.1. Implant Analog–Abutment Assembly and Specimen Embedding

A total of 48 implant analog–abutment assemblies were allocated to four groups (n = 12 per group) according to a 2 × 2 factorial design defined by the sealing material and superstructure material. Implant analogs compatible with the Straumann Tissue Level Regular Neck (RN; 4.8 mm shoulder diameter; Institut Straumann AG, Basel, Switzerland) system and RN synOcta cementable abutments (5.5 mm height, titanium; Institut Straumann AG, Basel, Switzerland), featuring an internal conical connection were used in this study. Straumann abutments are manufactured from commercially pure grade IV titanium, which provides high mechanical strength and fatigue resistance [8]; therefore, this system was preferred to ensure mechanical stability and standardized connection characteristics during testing. The abutments were connected to the implant analogs prior to embedment and were initially hand-tightened with minimal torque to ensure proper seating and facilitate handling during specimen preparation.

**Figure 1:**
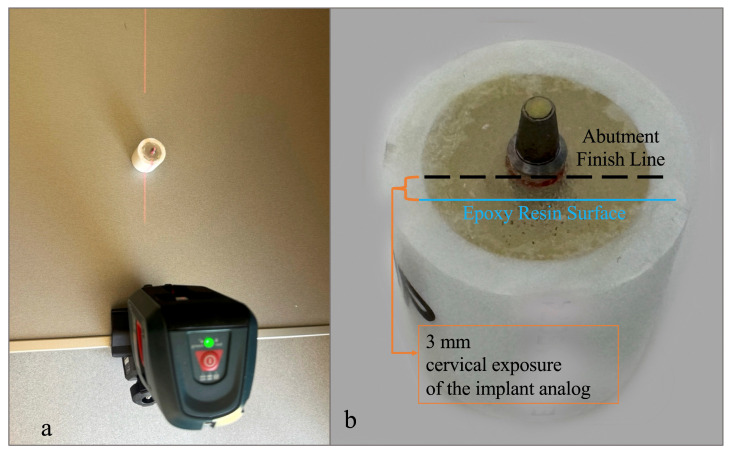
Implant analog–abutment embedding procedure: (**a**) alignment of the implant analog–abutment assembly using a line laser level during specimen embedding; (**b**) representative embedded specimen showing 3 mm cervical exposure of the implant analog above the epoxy resin surface, corresponding to the simulated bone loss condition.

Cylindrical polypropylene random copolymer (PPRC) molds with dimensions compatible with the chewing simulator (30 mm height, 25 mm outer diameter, 18 mm inner diameter) were used. The implant analog–abutment assemblies were embedded in epoxy resin model material (PL-2 and PLH-2; Vishay Precision Group Inc., Raleigh, NC, USA) using the molds as external housings. During embedding, a line laser level (Professional GLL 2; Robert Bosch Power Tools GmbH, Stuttgart, Germany) was projected along the abutment axis to ensure perpendicular alignment relative to the horizontal reference plane; the platform orientation was confirmed with a spirit level (Figure 1a).

The implant analogs were positioned with 3 mm of the cervical portion exposed above the resin surface. The exposed height was standardized using a digital caliper during embedding to allow complete visualization of the finish line during scanning and cementation procedures and to simulate the worst-case bone loss scenario described in ISO 14801:2016 [32] (Figure 1b). The resin was allowed to polymerize at room temperature for 24 h according to the manufacturer’s instructions.

### 2.2. Abutment Screw Tightening and Access Channel Sealing

**Figure 2:**
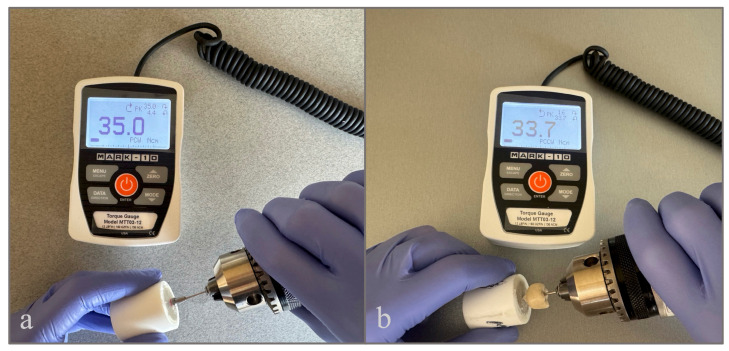
Torque measurement procedure: (**a**) tightening of the abutment screw to 35 Ncm using a digital torque gauge operating in peak clockwise mode; (**b**) measurement of the reverse torque value (RTV) in peak counterclockwise mode.

After complete polymerization, the abutment screws were tightened to 35 Ncm using a calibrated digital torque gauge (Model MTT03-12, TT03 Series; Mark-10 Corporation, Copiague, NY, USA) operating in peak clockwise mode (Figure 2a).

The screws were retightened to the same torque value after 10 min to compensate for the settling effect [7,14,29]. All torque applications were performed under standardized conditions using the same calibrated digital torque gauge with high measurement precision, and all measurements were conducted by a single operator to minimize variability and ensure consistency among specimens.

The screw access channels were sealed according to group allocation: 24 specimens were sealed with PTFE + composite and 24 with PVS putty. In the PTFE groups, an initial layer of PTFE tape was gently condensed over the screw head to provide isolation. A second PTFE layer of greater volume was then condensed to provide cushioning and facilitate retrievability. The thickness of the second layer was adjusted using a periodontal probe to ensure that 2 mm of composite resin remained coronally within the access channel [23,24]. A light-cured composite resin (Estelite Quick; Tokuyama Dental Corp., Tokyo, Japan) was applied to seal the remaining access channel and light-polymerized for 20 s using an LED curing unit (Curing Pen-E, model C-004-1, Changzhou Sifary Medical Technology Co., Ltd., Changzhou, China).

**Figure 3:**
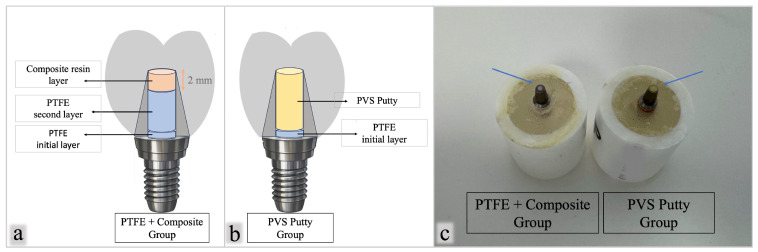
Screw access channel sealing protocols: (**a**) schematic representation of the PTFE + composite group; (**b**) schematic representation of the PVS putty group; and (**c**) representative specimens from both groups.

In the PVS groups, an initial PTFE layer of identical volume was condensed over the screw head to maintain screw access and retrievability. The remaining access channel was then filled with PVS putty (Elite HD+; Zhermack SpA, Badia Polesine, Italy) and allowed to polymerize according to the manufacturer’s instructions (Figure 3a–c). These sealing materials were selected to reflect conventional and alternative sealing strategies with different mechanical behavior, including PTFE combined with composite and a silicone-based PVS material, respectively.

### 2.3. Fabrication of Monolithic Zirconia and Resin Nano-Ceramic Crowns

Monolithic zirconia crowns (n = 24) were fabricated from pre-sintered zirconia blocks (Sigma Monolithic Zirconia; Sigmadent, Istanbul, Turkey). The restorations were designed using CAD software (Exocad; Exocad GmbH, Darmstadt, Germany; https://exocad.com; accessed on 18 April 2025) and milled with CAM software (WorkNC; Hexagon Manufacturing Intelligence, Saint-Aubin, France; https://hexagon.com; accessed on 18 April 2025) on a milling unit (Redon Hibrit; Redon Technology, Istanbul, Turkey) under dry conditions in standard milling mode (25,000 rpm). A standardized bur set (Redon Technology, Istanbul, Turkey), including a 2.0 mm milling bur, was used during fabrication for both groups. After milling, zirconia crowns were sintered according to the manufacturer’s instructions to achieve final mechanical properties.

Similarly, resin nano-ceramic crowns (n = 24) were milled from CAD/CAM blocks (Cerasmart 270; GC Corporation, Tokyo, Japan). The restorations were designed using the same CAD/CAM workflow as the zirconia group and were milled using a milling unit (Jiny Technology Co., Shenzhen, China) under wet conditions in standard milling mode (50,000 rpm). After milling, no sintering was required for Cerasmart, and no glazing procedures were applied prior to cementation in either group.

These restorative materials were selected to represent two clinically relevant approaches with distinct mechanical behaviors, namely a high elastic modulus ceramic (monolithic zirconia) and a hybrid resin-based ceramic (Cerasmart), thereby allowing evaluation of their influence on stress transmission within the implant–abutment system.

**Figure 4:**
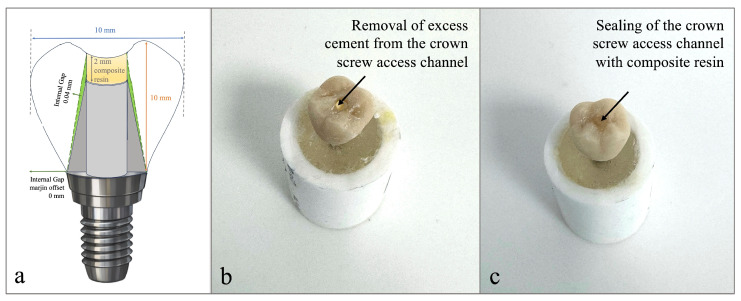
Crown design and cementation procedure: (**a**) schematic illustration of the standardized crown design, including internal gap and composite resin thickness; (**b**) removal of excess cement from the crown screw access channel after cementation; and (**c**) sealing of the crown screw access channel with composite resin.

All crowns were designed in a standardized maxillary molar form to simulate posterior clinical conditions. The total crown height, measured from the cusp tip to the cervical margin, was standardized at 10 mm, while the occluso-coronal distance between the occlusal surfaces of the abutment and crown was set at 2 mm to ensure uniform crown geometry. The occlusal table dimensions were set to 10 mm in both the mesiodistal and buccopalatinal directions. The occlusal morphology was identical across all specimens, with moderate cusp inclinations to reduce excessive lateral loading during cyclic testing. The internal gap (cement space) was set at 0.04 mm, and the margin offset at 0 mm for both restoration types to ensure complete marginal adaptation (Figure 4a).

A screw access channel with a diameter of 2 mm was incorporated into all crowns to allow access to the abutment screw during reverse torque testing. This configuration represents a screw-retrievable cement-retained design, in which the crown is cemented onto the abutment while maintaining screw access. This approach was selected to reflect a clinically relevant scenario, as it allows retrieval of the abutment screw when needed while preserving the esthetic advantages and passive fit associated with cement-retained restorations. In addition, this design enabled standardized access during RTV measurements without altering the prosthetic configuration.

### 2.4. Surface Treatment and Cementation Procedure

Prior to cementation, all abutments were airborne-particle abraded perpendicularly with 50 µm Al_2_O_3_ at 2 bar for 10 s from a distance of 10 mm using a laboratory sandblasting device (Twin-Pen Sandblaster VI, model JG-218, Wuhan Jinguang Medical Technology Co., Ltd., Wuhan, China), according to the manufacturer’s recommendations. The abutments were cleaned with alcohol and air-dried.

For the zirconia group, the internal surfaces of the crowns were additionally airborne-particle abraded under the same conditions, followed by application of an MDP-containing primer (Z-Prime Plus; Bisco, Schaumburg, IL, USA), which was allowed to react for 60 s and then gently air-dried for 10 s. No airborne-particle abrasion was performed on the internal surfaces of the resin nano-ceramic crowns.

A self-curing resin cement (Multilink Hybrid Abutment Cement; Ivoclar, Schaan, Liechtenstein) was applied as a thin layer to the internal walls of the crowns. The restorations were seated onto the corresponding abutments under controlled finger pressure, and complete seating was verified by confirming full adaptation at the finish line. Excess cement was removed, and the screw access openings were cleared before complete polymerization (Figure 4b).

Subsequently, the access channels were restored with the same composite material employed for sealing and light-cured using the same curing unit according to the manufacturer’s instructions. (Figure 4c). After cementation, all specimens were allowed to undergo complete polymerization at room temperature for 24 h before mechanical aging.

### 2.5. Thermomechanical Aging

**Figure 5:**
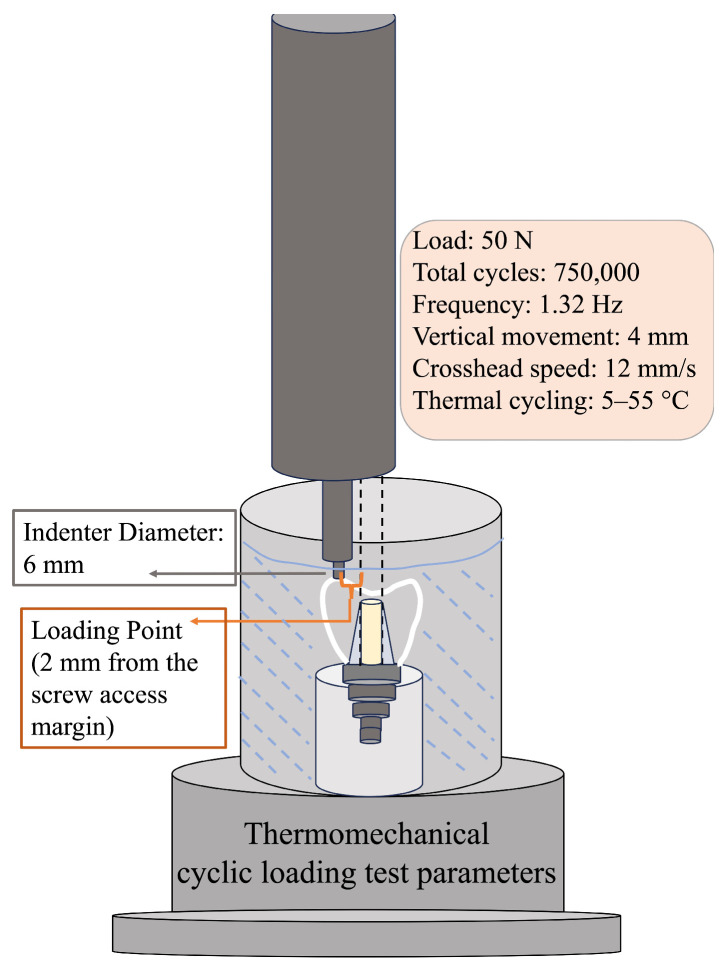
Schematic illustration of the thermomechanical cyclic loading setup, including the loading configuration, indenter position, and key testing parameters.

The specimens were then subjected to thermomechanical aging using a combined chewing simulator (Modental; Esetron, Ankara, Turkey). Each specimen was exposed to cyclic loading of 50 N for 750,000 cycles at a frequency of 1.32 Hz, with a vertical movement of 4 mm and a crosshead speed of 12 mm/s under soft-contact mode using a 6 mm diameter stainless steel antagonist indenter. The load magnitude of 50 N was selected to provide a controlled and reproducible fatigue model for evaluating screw joint stability and preload alterations without inducing catastrophic failure of the restorations. This loading level has been adopted in previous implant cyclic loading studies evaluating RTVs and preload maintenance [12,15] and is consistent with ISO 14801 fatigue testing principles, which do not prescribe a fixed load value but allow adjustment according to the experimental design [32]. Loading was applied at a point located 2 mm away from the screw access center to avoid contact with the composite-sealed area, generating an off-axis loading condition consistent with ISO 14801:2016 [32] (Figure 5).

Simultaneous thermal cycling was applied continuously between 5 °C and 55 °C throughout mechanical loading. Temperature conditions were maintained under controlled settings using the integrated thermal cycling unit throughout the testing period to ensure uniform and reproducible temperature exposure for all specimens.

### 2.6. Reverse Torque Measurement

After thermomechanical aging, the composite material located within the crown screw access channel was removed using a high-speed handpiece, and the sealing materials placed inside the access cavity were carefully retrieved to expose the abutment screw. RTVs were measured using the same calibrated digital torque gauge under standardized conditions by the same operator, with counterclockwise rotational force until initial screw loosening occurred (Figure 2b). The peak torque value at the moment of screw loosening was recorded in Ncm for each specimen.

### 2.7. Statistical Analysis

Statistical analyses were performed using the R software environment for statistical computing (version 4.3.2; R Foundation for Statistical Computing, Vienna, Austria). One specimen from the zirconia + PTFE group was excluded from the analysis due to screw stripping during reverse torque measurement. Descriptive statistics were calculated as mean ± standard deviation. The study followed a factorial design including two independent variables: superstructure material (monolithic zirconia or Cerasmart) and sealing material (PTFE + composite or PVS putty). Reverse torque values (RTV, Ncm) were analyzed using a two-way analysis of variance (ANOVA) to evaluate the main effects of these factors and their interaction. When significant differences were detected, pairwise comparisons were performed using Tukey’s honestly significant difference (HSD) post hoc test. Normality of the data distribution was assessed using the Shapiro–Wilk test, and homogeneity of variances was evaluated using Levene’s test. Effect sizes were calculated using eta-squared (η^2^). Effect sizes (η^2^) were interpreted according to Cohen’s guidelines, where 0.01 represents a small effect, 0.06 a medium effect, and 0.14 a large effect. In addition, torque loss (%) was calculated using the following formula [29,33]: Torque loss (%) = (35 − RTV)/35 × 100. The significance level was set at *p* < 0.05. In addition, 95% confidence intervals were calculated to provide a more comprehensive understanding of the data variability.

## 3. Results

**Figure 6:**
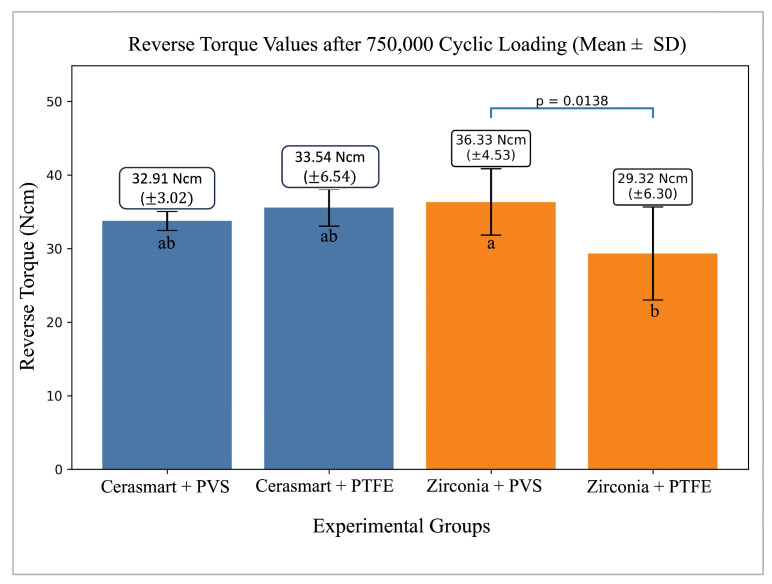
Reverse torque values (RTV) of the experimental groups after thermomechanical cyclic loading (mean ± standard deviation). Error bars represent 95% confidence intervals. Different lowercase letters indicate statistically significant differences between groups (*p* < 0.05, Tukey HSD test).

RTVs of the experimental groups are presented in Table 1 and Figure 6.

Among the experimental groups, the zirconia + PVS putty combination demonstrated the highest RTV (36.33 ± 4.53 Ncm), whereas the zirconia + PTFE group showed the lowest value (29.32 ± 6.30 Ncm). The Cerasmart groups exhibited comparable RTVs irrespective of the sealing protocol (Cerasmart + PVS putty: 32.91 ± 3.02 Ncm; Cerasmart + PTFE: 33.54 ± 6.54 Ncm). The groups were ordered as follows based on mean RTVs: zirconia + PVS putty, Cerasmart + PTFE, Cerasmart + PVS putty, and zirconia + PTFE.

**Figure 7:**
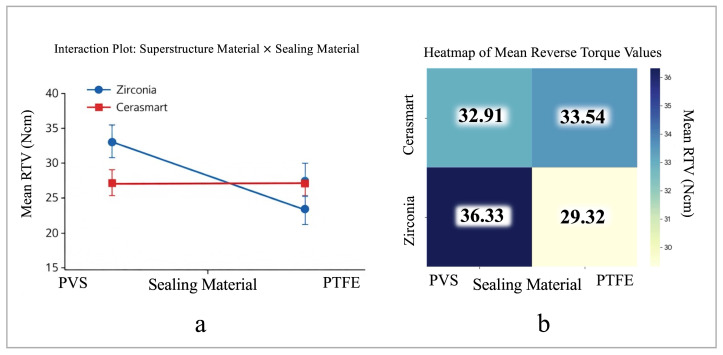
Interaction between superstructure material and sealing material on reverse torque values (RTV, Ncm): (**a**) interaction plot of mean RTVs with error bars representing 95% confidence intervals; and (**b**) heatmap showing the distribution of mean RTVs among the experimental groups.

Two-way ANOVA demonstrated that the main effect of superstructure material on RTV was not statistically significant (*p* = 0.8368). In contrast, the main effect of sealing material reached statistical significance (*p* = 0.0499). A statistically significant interaction was observed between superstructure material and sealing material (*p* = 0.0170) (Figure 7a,b).

The magnitude of this interaction corresponded to a moderate effect size (η^2^ = 0.116), according to Cohen’s classification, with approximately 11.6% of the variability in RTVs attributed to the combined influence of superstructure material and the access channel sealing protocol. This result reflects the contribution of the interaction effect in addition to statistical significance (Table 2).

Post hoc comparisons performed using Tukey’s HSD test revealed that the zirconia + PVS putty group exhibited significantly higher RTVs than the zirconia + PTFE group (*p* = 0.0138). No statistically significant differences were identified between the Cerasmart groups regardless of the sealing protocol (*p* > 0.05).

Analysis of torque loss (%) yielded results consistent with the RTV findings. Two-way ANOVA again revealed a significant interaction between superstructure material and sealing material (*p* = 0.0169). Tukey’s post hoc comparisons confirmed that torque loss was significantly lower in the zirconia + PVS putty group compared with the zirconia + PTFE group (*p* = 0.0138).

## 4. Discussion

The results of the present study demonstrated that the effect of the access channel sealing material on RTV depended on the superstructure material. Although the restorative material alone did not significantly influence RTV (*p* = 0.8368), a statistically significant interaction was observed between superstructure type and sealing protocol (*p* = 0.017, η^2^ = 0.116). The interaction between superstructure material and sealing protocol also demonstrated a moderate effect size, suggesting that the combined influence of these factors contributed meaningfully to the variability in RTVs. The influence of the sealing protocol was evident in zirconia restorations, whereas no significant differences were observed between the Cerasmart groups. The inclusion of effect size measures alongside *p*-values provides a more comprehensive interpretation of the clinical relevance of the findings, as statistically significant differences may not always reflect meaningful biomechanical effects. This finding may be explained by the lower elastic modulus of resin-based materials such as Cerasmart, which may absorb part of the applied load and transmit lower stresses to the implant–abutment complex. A similar biomechanical explanation has been reported by Kaleli et al. (2018), who demonstrated reduced implant–abutment stresses when using PEEK abutments with a comparably low elastic modulus [34]. Likewise, another study has reported that the use of alternative hybrid abutment materials, such as PEEK and lithium disilicate rather than zirconia, may reduce the risk of screw loosening [26]. Although the cited studies investigated abutment materials rather than superstructures, their findings regarding stress transmission may provide a useful biomechanical perspective for interpreting the potential influence of restorative materials on screw stability. Taken together, these findings suggest that the observed interaction between superstructure material and sealing protocol may be attributed to differences in elastic modulus, stress dissipation behavior, and energy absorption capacity within the system, where materials with a higher elastic modulus tend to transmit functional loads more directly to the implant–abutment interface, increasing the sensitivity of the system to the sealing condition, whereas more elastic materials may absorb, dissipate, and partially dampen functional stresses, exhibiting a cushioning effect that reduces stress concentration at the screw joint. In addition, differences in mechanical rigidity between the restorative material and the sealing approach may influence load distribution and micro-movement at the implant–abutment interface, further contributing to the observed interaction effect.

Zirconia is considerably stiffer and may transfer occlusal forces more directly to the screw joint [31], which may explain the greater influence of the sealing protocol observed in the zirconia groups. Supporting this biomechanical concept, Aboelkhier et al. (2025) reported that restorative materials with higher elastic modulus values, such as zirconia (~220 GPa), exhibited greater torque loss compared with lithium disilicate (~64 GPa) and polymer-infiltrated ceramic (~35 GPa) [35]. In contrast, Cerasmart, a resin-nanoceramic material composed of a highly filled resin matrix with dispersed ceramic nanoparticles, has a considerably lower elastic modulus (≈18–20 GPa), which may allow greater stress absorption and reduce stress transmission to the implant–abutment complex. However, Delben et al. (2014) reported that, in screw-retained implant-supported crowns, detorque reduction occurred irrespective of the veneering material used (ceramic or resin), and no material demonstrated clear superiority in maintaining screw preload [15]. This discrepancy may be related to differences in prosthetic retention design between screw-retained and cement-retained restorations. Similarly, Al-Zordk et al. (2020) found no significant differences in torque maintenance among zirconia, lithium disilicate, and PEEK hybrid-abutment restorations, possibly due to the use of thermal aging alone without mechanical cyclic loading [31]. However, studies specifically evaluating the influence of superstructure materials on RTVs remain limited. For this reason, two restorative materials with different compositions and biomechanical behavior were selected in the present study. These materials were deliberately selected to represent clinically relevant restorative approaches with distinct elastic moduli and mechanical responses. Monolithic zirconia was included as a widely used high-strength ceramic, whereas the hybrid resin-based ceramic Cerasmart represents a more recently introduced material with enhanced energy absorption and stress-dissipating characteristics. This selection allowed not only comparison between conventional and contemporary materials but also evaluation of how differences in mechanical behavior may influence load transmission and screw joint stability within the implant–abutment system, as well as how these differences may interact with the behavior of access channel sealing materials under functional loading conditions.

The findings of the present study also suggest that the type of access channel sealing material may influence the stability of the abutment screw. PTFE is widely used as an access channel sealing material due to its ease of manipulation, retrievability, protection of the screw head, and cushioning properties [22]. PVS materials, composed of hydrophobic polydimethylsiloxane polymers, may also serve as sealing materials for screw access channels. In the zirconia groups of the present study, sealing the access channel with PVS putty resulted in higher RTVs compared with PTFE. This finding may be attributed to the elastic and cushioning characteristics of silicone-based materials, which may help distribute stresses within the access channel and reduce direct load transmission to the screw joint. Consistent with this observation, Zhou et al. (2022) evaluated different materials for sealing the screw access channel and reported that PVS demonstrated superior microleakage sealing efficacy and favorable removal characteristics compared with PTFE [36]. Similarly, silicone-based sealing agents such as GapSeal applied at the implant–abutment interface have been shown to improve torque maintenance after cyclic loading [7], which may support the favorable performance of silicone-based materials observed in the present study. In the present study, the sealing materials were selected to represent different approaches, including the widely used PTFE combined with composite as a conventional sealing strategy and PVS as a silicone-based alternative with distinct elastic and stress-dissipating characteristics. This selection allowed evaluation of how different sealing behaviors may influence load transmission and screw joint stability under functional conditions.

From a clinical perspective, silicone-based materials such as PVS may be particularly advantageous for access channel sealing in situations where stress distribution within the access channel is critical, such as in restorations subjected to high occlusal loads or parafunctional loading conditions such as bruxism, as well as in cases involving superstructure materials with a high elastic modulus. In such scenarios, the elastic behavior of PVS may help absorb functional stresses and support the preservation of screw joint stability over time, suggesting that the selection of access channel sealing materials should be selected not only based on retrievability but also considering their potential biomechanical influence on implant–abutment stability. Conventional approaches such as PTFE combined with composite resin may still provide acceptable clinical performance; however, their relatively more rigid behavior may result in different stress transmission characteristics compared with silicone-based materials.

Although implant–abutment screw stability and preload maintenance are recognized as clinically important factors influencing the long-term success of implant-supported restorations, direct clinical evidence specifically evaluating RTVs remains limited due to methodological and ethical constraints associated with in vivo measurements. Therefore, most available data are derived from in vitro studies simulating functional loading conditions [19,31,35,36]. Nevertheless, clinical studies have consistently reported that screw loosening is a common mechanical complication in implant-supported restorations, supporting the clinical relevance of investigating factors that may influence preload maintenance under controlled experimental conditions [1,3,4,5].

In contrast, PTFE combined with composite forms a relatively rigid seal, which may transmit functional forces more directly to the abutment screw. While previous studies have mainly investigated screw access channel sealing materials in terms of retention of cement-retained restorations or microleakage [21,22,36], the present study focused on their potential influence on abutment screw stability by evaluating RTV after cyclic loading. In a clinical study evaluating materials used to restore the occlusal screw access opening of screw-retained implant crowns, both a nano-hybrid composite resin and a self-curing resin demonstrated comparable marginal integrity and wear behavior after 12 months of clinical function [37]. However, that study primarily evaluated the surface durability of the occlusal sealing material rather than its potential influence on abutment screw stability, which was the primary outcome investigated in the present study.

Implant–abutment connection design and preload are recognized as important mechanical factors influencing the stability of the implant–abutment interface [33]. In the present study, cement-retained restorations were supported by Straumann RN synOcta titanium abutments with an internal octagonal anti-rotational connection. Internal implant–abutment connections have been reported to enhance joint stability by improving mechanical interlocking and directing functional loads to the internal implant walls rather than directly to the retention screw, thereby reducing micromovement and supporting preload maintenance [33,38]. Similarly, Jorge et al. (2013) reported that internal Morse taper connections exhibit lower torque loss (32.88%) after mechanical cyclic loading compared with external hexagon designs [10]. Accordingly, an internal implant–abutment connection was selected in the present study to provide a stable mechanical configuration and to allow a clearer evaluation of the effects of restorative material and access channel sealing on screw stability. In this context, the interaction observed between superstructure material and sealing protocol in the present study may provide additional insight into the factors influencing screw stability under simulated functional loading conditions.

The abutment used in this study had a height of 5.5 mm and a platform diameter of 4.8 mm, which may provide a relatively deep internal screw access channel capable of accommodating a greater volume of sealing material and thereby enhancing its cushioning capacity, potentially influencing stress transmission to the abutment screw. Supporting this interpretation, Moris et al. (2015) compared abutments with platform diameters of 4.8 mm and 3.8 mm and reported greater failure rates and higher torque loss after mechanical cycling in the narrower abutment group, whereas the wider 4.8 mm abutments demonstrated improved mechanical stability [29].

Prosthetic retention design may also influence load transfer within the implant–abutment complex. In cement-retained restorations, the cement layer may contribute to the absorption and distribution of occlusal forces across the restoration–abutment–implant interface, potentially reducing stress concentration at the screw joint, whereas screw-retained designs may transmit occlusal forces more directly to the connection interface, particularly under lateral loading conditions, consistent with the observations reported by Bishti et al. [3]. Chen et al. (2023) evaluated angulated screw channel abutments (0°, 15°, and 25°) and reported that increasing screw channel angulation may negatively influence torque maintenance after cyclic loading [19]. However, other studies have reported no significant differences in RTV between different screw channel angulations or between straight and angled screw-retained restorations [39,40]. Similarly, Kim et al. (2009) reported comparable RTV between screw-retained and cement-retained prostheses after cyclic loading, suggesting that prosthetic retention design alone may not significantly influence abutment screw stability [20]. Accordingly, straight cement abutments were selected in the present study to minimize potential biomechanical confounding factors and to allow clearer interpretation of the effects of restorative and sealing materials on screw stability. Within this framework, a screw-retrievable cement-retained configuration was adopted, allowing access to the abutment screw while preserving the advantages of cement-retained restorations. This approach reflects a clinically relevant scenario in which retrievability may be required for maintenance or complication management, while maintaining favorable esthetic outcomes and load distribution. Compared with fully cement-retained designs, this configuration allows access to the abutment screw without destruction of the restoration, whereas compared with fully screw-retained designs, it preserves the benefits of a cement interface, including improved passive fit and stress distribution. Furthermore, this configuration enabled controlled and repeatable access to the abutment screw during RTV measurements without modifying the prosthetic assembly, thereby enhancing the methodological consistency of the study.

Cyclic loading tests, with frequencies ranging between 1 and 19 Hz, are widely used to simulate functional masticatory forces [33]. An 8-year follow-up study reported that all complications in 249 implant-supported fixed prostheses occurred within the first 10 months of function [41], supporting the use of 500,000 to 1,000,000 cycles in masticatory simulation to reproduce clinically relevant fatigue effects [20]. Thermal cycling may also influence the mechanical behavior of the implant–abutment complex by promoting degradation of the cement layer, altering the mechanical response of restorative materials, and contributing to fatigue processes within metallic components. In the present study, specimens were subjected to 750,000 thermomechanical loading cycles, a protocol commonly considered to reflect approximately three years of clinical function.

Previous studies have applied eccentric loading conditions at distances of up to 5 mm from the abutment center [42,43], and it has been reported that eccentric lateral loading may result in higher RTVs compared with centric loading [44]. The occlusal load in the present study was applied 2 mm lateral to the screw access margin (3 mm from the screw access center), creating an off-axis loading condition and generating a bending moment within the implant–abutment complex, which is consistent with the off-axis loading concept described in ISO 14801:2016 for fatigue testing of dental implants [32]. This configuration was also adopted to avoid direct contact with the composite-sealed access channel while allowing evaluation of stress transmission through the restorative materials. Such off-axis loading may partly explain why the influence of the sealing protocol was more evident in the zirconia groups, whereas no significant differences were observed between the Cerasmart groups in this study. The torque loss analysis yielded findings consistent with the RTV results, indicating that the interaction between restorative material and sealing protocol was similarly reflected in both measurements and supporting the reliability of the observed mechanical behavior.

RTV is commonly used as an indirect indicator of residual preload within the implant–abutment screw joint. A reduction in preload and frictional resistance at the contacting surfaces generally results in lower RTVs [14]. Following the initial tightening of the abutment screw, a reduction in preload may occur due to the settling effect associated with microscopic irregularities on the contacting surfaces of implant components. During the early moments after torque application, deformation and adaptation of these micro-irregularities may occur, leading to a small loss of preload, a phenomenon commonly referred to as embedment relaxation [10]. For this reason, clinical protocols often recommend retightening the abutment screw several minutes after the initial torque application. Surface adaptation after tightening may reduce the applied torque by approximately 2–10% [45], whereas torque losses of up to 24.8% have also been reported [46]. Consequently, the RTVs obtained after cyclic loading in the present study may reflect the ability of different restorative and sealing materials to influence preload maintenance at the implant–abutment interface. In this context, the observed difference in RTVs, particularly the 7.01 Ncm higher RTV in the zirconia groups sealed with PVS (36.33 Ncm) compared with PTFE (29.32 Ncm), may be considered clinically relevant, as higher RTVs are generally associated with improved maintenance of preload and a reduced risk of screw loosening. This effect may be more pronounced in zirconia restorations, where the higher elastic modulus may lead to more direct stress transmission to the implant–abutment interface, thereby increasing the sensitivity of the system to the sealing protocol. However, this potential clinical significance should be interpreted with caution, as in vitro conditions may not fully replicate the complex biomechanical environment encountered in vivo.

Appropriate tightening torque is critical for maintaining long-term screw stability in implant-supported restorations, as insufficient preload may lead to loss of clamping force and subsequent mechanical or biological complications at the implant–abutment interface [3]. Therefore, the use of a calibrated torque-controlling device is essential to ensure reliable preload [11]. In the present study, both the initial tightening torque and the peak RTV were measured using a calibrated digital torque gauge, ensuring precise torque application and reliable preload assessment. Notably, RTVs exceeding the initial tightening torque of 35 Ncm were observed in some specimens, particularly in the zirconia + PVS putty group. Although this finding may appear counterintuitive, it has been previously reported and can be explained by the combined effects of settling, also referred as embedment relaxation, and frictional changes at the implant–abutment interface following cyclic loading [15,47]. Mechanical cycling may lead to plastic deformation and improved adaptation of micro-irregularities at the contacting surfaces, thereby increasing the real area of contact and enhancing interfacial friction. As a result, greater resistance to screw loosening may be observed, leading to RTVs that exceed the initially applied torque.

The findings of the present study partially supported the study hypothesis, as the access channel sealing material showed a significant effect on RTVs, whereas the superstructure material alone did not significantly influence RTVs after cyclic loading. However, a significant interaction between restorative material and sealing protocol was observed, indicating a material-dependent effect, whereby the influence of the sealing protocol varied according to the type of restorative material used.

The limitations of the present study should be considered when interpreting the results. It should be emphasized that the applied cyclic loading protocol represents a simplified in vitro simulation of clinical conditions and does not fully reproduce the magnitude, complexity, and biomechanical environment of functional forces encountered in the posterior region. Although the loading magnitude was lower than peak masticatory forces, it was intentionally selected to provide a controlled fatigue model without leading to structural compromise of the restorations. Therefore, the mechanical responses observed in this study may not fully reflect clinical conditions, particularly under higher functional loads or long-term service. In addition, the present study included one implant system with an internal connection and straight cementable abutments. Accordingly, the observed interaction between restorative material and sealing protocol may vary with different implant systems or connection geometries. While implant analog–abutment assemblies are commonly used as a practical alternative to clinical implants due to the requirements of laboratory-based experimental setups and the relatively large number of specimens, it should be acknowledged that they represent an experimental approximation of clinical conditions, and that differences in material properties and alloy composition may influence load transmission, potentially affecting screw stability. In this context, it should be considered alongside the other limitations when interpreting the present findings. Furthermore, a single crown configuration and loading geometry were used in this study, which may not fully represent the variability of clinical occlusal conditions. A single type of luting cement was used for crown fixation, and the evaluation was limited to two restorative materials and two access channel sealing protocols under controlled laboratory conditions. Moreover, the thermomechanical loading protocol simulated approximately three years of clinical function; therefore, the long-term stability of the observed effects should be interpreted with caution, as different fatigue durations or loading conditions may lead to different outcomes.

Future studies are needed to further investigate the clinical performance of different access channel sealing materials under varying loading conditions, implant systems, and connection geometries. In addition, evaluating alternative materials with different mechanical properties, as well as variations in access channel configuration and prosthetic design, may provide deeper insight into their influence on implant–abutment stability. Future research may also focus on the combined effects of restorative material properties and sealing strategies under more complex and dynamic loading conditions that better simulate clinical reality. Furthermore, clinical studies are required to validate these in vitro findings and to assess their applicability in real intraoral environments.

## 5. Conclusions

Within the limitations of this in vitro study, abutment–implant screw stability after thermomechanical cyclic loading was influenced by the interaction between the superstructure material and the access channel sealing protocol. RTVs ranked from highest to lowest as follows: zirconia + PVS putty, Cerasmart + PTFE, Cerasmart + PVS putty, and zirconia + PTFE. Although the restorative material alone did not significantly affect RTV, a material-dependent effect of the sealing protocol was observed, indicating that the influence of the sealing strategy on screw stability depended on the mechanical behavior of the superstructure material.

In particular, zirconia restorations sealed with PVS putty demonstrated significantly higher RTVs and lower torque loss compared with the zirconia + PTFE + composite configuration. This finding suggests that more elastic silicone-based sealing materials may contribute to improved stress distribution within the screw access channel and may help preserve preload under functional loading conditions in rigid restorative materials. In contrast, resin nano-ceramic restorations, such as Cerasmart, showed comparable RTVs regardless of the sealing protocol, indicating that the mechanical behavior of more resilient restorative materials may partially mitigate the influence of the access channel sealing configuration.

Taken together, these findings highlight that implant-supported restorations should be considered as a biomechanical system in which restorative material properties and access channel sealing strategies interact to influence screw joint stability. From a clinical perspective, careful selection of the access channel sealing material may be particularly relevant in rigid restorative materials such as zirconia, where sealing configuration may affect the maintenance of abutment screw preload under functional loading.

## Figures and Tables

**Table 1:** Reverse torque values (RTV) of the experimental groups according to restorative material and sealing material (mean ± standard deviation, Ncm).

Restorative Material	Sealing Material	RTV (Mean ± SD, Ncm)
Cerasmart	PVS	32.91 ± 3.02 ^ab^
Cerasmart	PTFE	33.54 ± 6.54 ^ab^
Monolithic Zirconia	PVS	36.33 ± 4.53 ^a^
Monolithic Zirconia	PTFE	29.32 ± 6.30 ^b^

RTV, reverse torque value; SD, standard deviation; PVS, polyvinyl siloxane; PTFE, polytetrafluoroethylene. Different superscript letters indicate statistically significant differences between groups (*p* < 0.05, Tukey HSD test).

**Table 2:** Effect size (η^2^) values for the main effects and interaction.

Factor	Eta-Squared (η^2^)	Effect Level
Superstructure Material	0.0008	Very small/negligible
Sealing Material	0.0764	Moderate effect
Interaction (Material × Sealing)	0.1158	Moderate to large effect

η^2^, eta-squared effect size; interpreted according to Cohen’s guidelines.

## Data Availability

The data presented in this study are available on request from the corresponding author due to their potential use for future research purposes.

## Abbreviations

The following abbreviations are used in this manuscript:

PTFE	Polytetrafluoroethylene
PVS	Polyvinyl siloxane
PEEK	Polyetheretherketone
RTV	Reverse torque value
ANOVA	Analysis of variance
HSD	Honestly significant difference
SD	Standard deviation
CAD	Computer-aided design
CAM	Computer-aided manufacturing
